# Voxel-based morphometry predicts shifts in dendritic spine density and morphology with auditory fear conditioning

**DOI:** 10.1038/ncomms8582

**Published:** 2015-07-07

**Authors:** O. P. Keifer Jr, R. C. Hurt, D. A. Gutman, S. D. Keilholz, S. L. Gourley, K. J. Ressler

**Affiliations:** 1 Department of Psychiatry and Behavioral Sciences, Emory University School of Medicine, Atlanta, Georgia 30329, USA.; 2 Yerkes National Primate Research Center, Atlanta, Georgia 30329, USA.; 3 Department of Biomedical Informatics, Emory University School of Medicine, Atlanta, Georgia 30322, USA.; 4 Department of Neurology, Emory University School of Medicine, Atlanta, Georgia 30322, USA.; 5 Department of Biomedical Engineering, Emory University School of Medicine, Atlanta, Georgia 30322, USA.; 6 Department of Pediatrics, Emory University School of Medicine, Atlanta, Georgia, 30322, USA.; 7 Howard Hughes Medical Institute, Bethesda, Maryland 20815, USA.

## Abstract

Neuroimaging has provided compelling data about the brain. Yet the underlying mechanisms of many neuroimaging techniques have not been elucidated. Here we report a voxel-based morphometry (VBM) study of *Thy*1-YFP mice following auditory fear conditioning complemented by confocal microscopy analysis of cortical thickness, neuronal morphometric features and nuclei size/density. Significant VBM results included the nuclei of the amygdala, the insula and the auditory cortex. There were no significant VBM changes in a control brain area. Focusing on the auditory cortex, confocal analysis showed that fear conditioning led to a significantly increased density of shorter and wider dendritic spines, while there were no spine differences in the control area. Of all the morphology metrics studied, the spine density was the only one to show significant correlation with the VBM signal. These data demonstrate that learning-induced structural changes detected by VBM may be partially explained by increases in dendritic spine density.

## Abstract (web summary)

Voxel-based morphometry (VBM) involves comparisons of high resolution structural images of the brain between groups, but what causes changes in the VBM signal is unclear. Here the authors perform a VBM study of Thy1-YFP mice following auditory fear conditioning and propose that the signal changes can be partially explained by increases in dendritic spine density.

## Graphical abstract

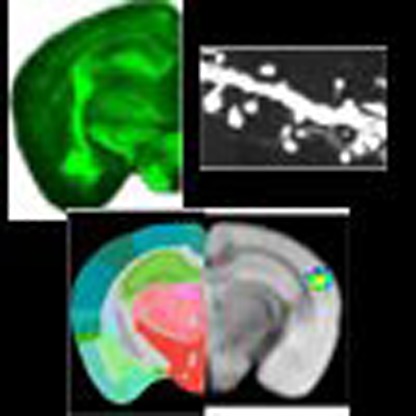

The use of various magnetic resonance imaging (MRI) techniques (for example, functional MRI, voxel-based morphometry (VBM) and diffusion-tensor imaging) to understand human neurological physiology and pathology is now ubiquitous. With the exception of functional MRI^1^, few of these methods have undergone rigorous study to determine the underlying causes for signal or signal change^2^,^3^. For example, VBM is a process by which high-resolution structural volumes of brains undergo statistical comparison to draw conclusions about differences in brain regions between groups^4^. As part of the VBM analysis, the brain is segmented into different components reflecting grey matter, white matter and cerebral spinal fluid. Focusing on grey matter analysis, the metric used for comparison in VBM depends in part on how ‘grey' an area is based on this segmentation with adjustments for registration into a standard space. The term ‘grey matter density (GMD)' is applied to this measure, and presumably differences in GMD represent an underlying shift in the makeup of grey matter in that brain area.

However, there is no current evidence for what changes occur in the grey matter to account for changes in the VBM signal (for example, GMD). This lack of a mechanistic understanding is a considerable gap in our current knowledge when considering that VBM is widely used as a research tool. For example, in the neuropsychiatric literature, a number of fear-related disorders, such as post-traumatic stress and panic disorder, among others, have been associated with regional alterations using VBM^5^,^6^,^7^,^8^. Even more broadly, there are a large number of influential papers that have shown effects using VBM-based techniques^3^,^9^,^10^,^11^,^12^. These studies point to the tremendous advantage of VBM as an *in vivo*, non-invasive analysis of human brains in health and disease. However, as a natural consequence of human-focused research, there is only speculation regarding what cellular-level changes underlie the structural imaging signal^13^. Hypotheses about the mechanisms associated with the VBM signals include, but are not limited to, altered neurogenesis and glial proliferation, changes in neuronal or glial size, angiogenesis and endothelial cell proliferation, and shifts in dendritic spine size and density.

As aforementioned, the main hindrance in studying the underlying mechanisms of MRI-based signal changes is the frequent application to human studies, but its relatively sparse use in animal model studies. This is surprising to some degree since the translational potential of MRI studies is unparalleled. On the basis of this utility and in a partial readdress, our previous work has focused on developing *ex vivo* Diffusion Tensor Imaging (DTI)-based imaging in the mouse model, which has allowed for the development of an imaging and analysis protocol to examine mechanisms of VBM-based structural change in this study^2^,^14^,^15^. We use these tools to address some of the potential cellular mechanisms that may account for the changes in brain structure as detected by VBM. Notably, our selection of *ex vivo* imaging is based on its utility in ascertaining high-resolution, high-contrast and more precise imaging of brain volumes, thereby allowing for the best possible analysis of anatomical changes^16^.

Since VBM is most often used for a comparison between two patient populations, we elected to use auditory fear conditioning as our experimental model. Fear conditioning is a robust paradigm used worldwide to study mechanisms of emotional memory formation and regulation^17^. Although a tremendous amount of work has focused on amygdala-dependent mechanisms of fear conditioning, fewer studies have examined the neural mechanisms underlying structural plasticity within cortical sensory areas associated with paradigms such as auditory fear conditioning^18^,^19^. As such, we used our high-throughput *ex vivo* imaging protocol to acquire T2 high-resolution structural volumes of *Thy*1-YFP-expressing transgenic mice that underwent an auditory fear conditioning paradigm. Following the acquisition of high-resolution scans, a subset of the mouse brains were then sectioned and prepared for confocal imaging. The metrics of confocal analyses focused on the proposed mechanisms of VBM signal change, including cortical thickness, layers V and VI neuron diameter, dendritic spine density, spine width, spine length, the ratio of width to length, and cell nuclei width and density. Of these measurements, several were then correlated to the VBM signal to draw preliminary conclusions about the cellular mechanisms underlying VBM.

Remarkably, we found that there were increases in the VBM signal in the auditory cortex (AC), several nuclei of the amygdala and the insula—areas previously strongly associated with fear conditioning, but without prior VBM data in mice models. Further, proceeding with the confocal analysis of the AC, of the metrics evaluated, the auditory fear conditioning group had a higher density of wider, although shorter, dendritic spines. Importantly, the density of spines significantly correlated with increases in auditory fear conditioning-induced VBM signal in the AC. There were no significant differences in the layers V and VI neuron soma diameters, nuclei densities or widths between the groups. Further, there was also no significant change in the AC cortical thickness. In addition, analysis of the rhinal cortex, which was selected as the control region, revealed no significant difference in the VBM analysis or the confocal analysis. Therefore, by showing a significant correlation between spine density and VBM signal in the AC, these results address the tremendous gap in our current understanding of the cellular underpinnings of VBM. In addition, they highlight the feasibility and large potential for the emerging field of small-animal MRI imaging as a scientifically and translationally powerful tool.

## Results

### Auditory fear conditioning

**Figure 1:**
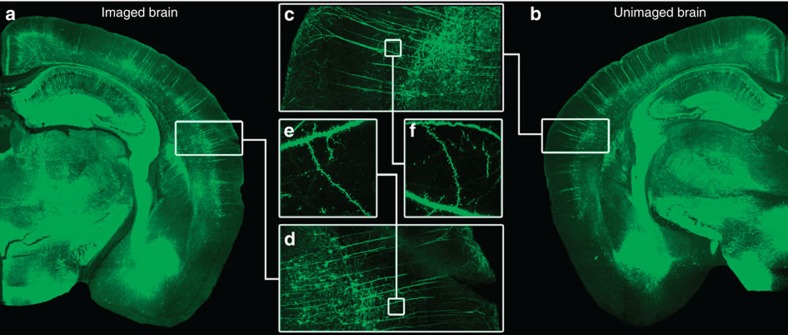
No apparent differences magnetic resonance imaged and naive mouse. (**a**,**b**) Representative composite images of the *Thy*1-YFP-expressing neurons for a mouse that underwent the training, perfusion, embedding, MRI, and then confocal sectioning and analysis versus one that only underwent perfusion and confocal sectioning. (**c**,**d**) These images show a magnified composite section of the AC for both the magnetic resonance imaged and naive brain. (**e**,**f**) Selection of a specific dendritic length to show no difference in the signal and imaging quality between brains having undergone the MRI embedding procedure and those that had not.

**Figure 2:**
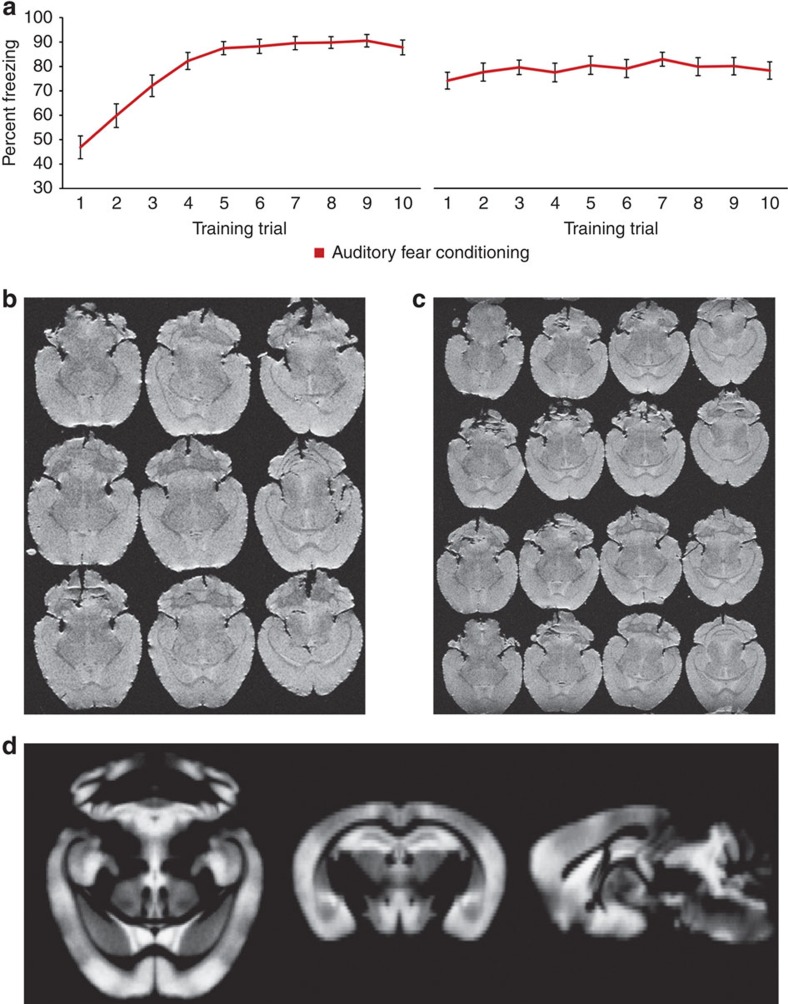
Fear conditioning and high-throughput *ex vivo* structural MRI. (**a**)The two graphs illustrate the acquisition of auditory fear conditioning (illustrated with per cent total freezing during the tone-conditioned cue during each training trial) over the course of 5 days (days 1 and 5 shown). The left graph shows the acquisition of fear during the first day as indicated by the increased freezing behaviour during the presentation of the tone. The right graph shows the freezing behaviour of the group during the last day of training, which has plateaued at ∼80% freezing during the presentation of the tone. (**b**,**c**)The middle two images show representative images of the T2 RARE high-resolution *ex vivo* acquisition of both 9 (**b**) and 16 brains (**c**) based on our *ex vivo* technique. (**d**)The result of segmenting the grey matter from each mouse MRI volume to create a grey matter skeleton template (*n*=54 brains) used in the present study.

To facilitate comparisons of the VBM results with underlying cellular measures, *Thy1*-YFP-expressing mice were selected as the experimental model. *Thy1*-YFP-expressing mice have robust fluorescent signals (Fig. 1)—most notably in cortical layer V pyramidal neurons followed by layer VI neurons^20^. Given the role of sensory cortex in memory^21^,^22^,^23^ and our interest in long-term structural changes underlying a long-term emotional memory, our auditory fear conditioning paradigm follows previous work related to fear incubation^24^. Concurrently, prior evidence from our lab suggests that a number of immediate-early and synaptic plasticity genes are expressed in the sensory cortex during the consolidation period following auditory fear conditioning^25^. In the current studies, *Thy1*-YFP-expressing mice underwent 5 days of conditioning with 10 tone-shock pairings each day. The resulting fear acquisition curves are presented in Fig. 2a for days 1 and 5. The curves show the expected acquisition of freezing across days, suggesting that the animals acquired and maintained the pairing of the tone with the electrical shock. The control group was handled identically except tones and shocks were withheld.

### Voxel-based morphometry analysis

Two weeks after training, mice were placed in deep anaesthesia, were perfused with 4% formaldehyde in phosphate-buffered saline, and the brains were extracted and were postfixed overnight. The brains were then inspected for any signs of damage and were then embedded into an agarose-gadolinium (III) oxide matrix for our high-throughput *ex vivo* imaging method^2^,^14^,^15^. This method allows for the simultaneous scanning of nine brains (as used for this study), Fig. 2b, although we recently have been able to acquire 16 brains at a time Fig. 2c). The two groups (*n*=27 per group) were equally distributed through six rounds of high-resolution RARE T2-weighted image acquisitions on a Bruker 9.4 Tesla scanner. All MRI brain volumes were examined for any signs of artefact in the cortex.

**Figure 3:**
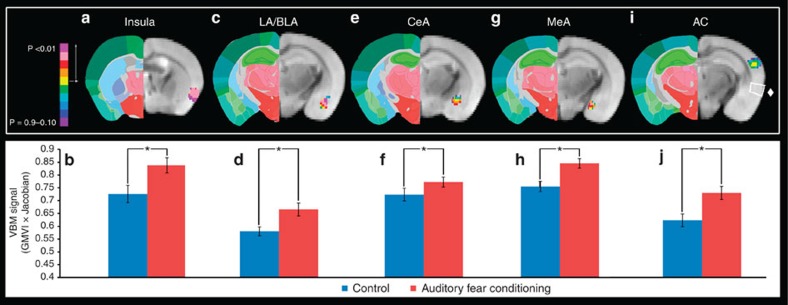
VBM findings in the amygdala, AC and insula. (**a**) The results of the VBM analysis showing a significant difference in the insular cortex between the auditory fear conditioning (AFC) and cage-handled control group (*n*=27auditory fear conditioning, *n*=27 controls). (**b**) The bar graphs show the quantification of the VBM signal (grey matter voxel intensity (GMVI) multiplied by the Jacobian) sampled from the voxels of the insular cortex that were significantly different. The analogous data are shown for the LA and BLA in **c**,**d**, the CeA in **e**,**f**, the MeA in **g**,**h** and the AC in **i**,**j**, in addition **i** shows a white box around the rhinal cortex marked by a ♦, which was not significantly different between groups. Data are presented as mean±s.e.m. *P≤0.05.

Since each MRI volume contained nine brains, the first step was parsing out each brain into its own individual volume. All 54 brains were then processed through the VBM analysis package available in the FSL Imaging Analysis Suite comparing the auditory fear-conditioned group with the cage-handled control. As a component of this analysis, the brains were segmented into white matter and grey matter. The grey matter skeleton of the 54 brains is presented in Fig. 2d. On the basis of the auditory fear conditioning literature, several cortical areas were investigated in an *a priori* manner. Masks were created that were inclusive of the region of interest including the infralimbic (IL) and prelimbic (PL) cortices, insular cortex, AC, medial, basolateral/lateral and central amygdala (MeA, BLA/LA and CeA, respectively), anterior cingulate cortex (ACC)/retrosplenial cortex and a control region of the rhinal cortex. Of these areas, the AC, MeA, CeA, BLA/LA and insula showed significant increases in the auditory fear conditioning group (Fig. 3). Notably, of the brain regions investigated here, there were no cases where there were significant decreases in the VBM signal for the auditory fear-conditioned group. Further, the rhinal cortex, which served as a control region, as well as the *a priori* hypothesized IL and PL cortices, never reached significance, nor did they trend towards significant differences. Of the areas showing significant differences, the AC was selected for further analyses since the *Thy1*-YFP mice have strong fluorescent expression in the AC allowing for examination and analysis of neuronal morphology, such as layers V and VI soma diameter and dendritic spine density, width and height (for example, as seen in Fig. 1, the different nuclei of the amygdala have either very little fluorescence expression or have very dense fluorescence expression prohibiting neuronal analysis of individual neurons).

### Dendritic spine density, head width and length

To understand the underlying cellular mechanisms that may account for the VBM changes in the AC, after the acquisition of the MRI volumes, the same brains were then unembedded and sectioned into 35 μm sections on a Microm HM450 freezing sliding microtome. A preliminary comparison with fluorescence microscopy of a post-MRI brain to a brain that did not undergo embedding and imaging (Fig. 1a–f) ensured that the embedding methodology did not compromise the cellular integrity or fluorescence signal. Of the 54 brains imaged and selected for VBM analysis, we randomly sampled a third of the brains from each group (nine brains) for microscopy analysis based on previous work^26^,^27^,^28^.

Of the cortical areas that were significantly different between groups in the VBM portion of the study, we selected to further analyse the AC. The AC was advantageous in many ways including its relative size in the coronal plane (allowing for a larger collection area), the ease of identification on coronal sections relative to known anatomical landmarks, and the density and intensity of the *Thy1*-YFP neurons in that portion of the cortex. The confocal analyses focused on several neuronal features including the second- and third-order dendrites of the layer five pyramidal neurons, which allowed for the quantification of dendritic spine density, length and head diameter. Further, the confocal analysis allowed for the measurements of the soma diameter for layers V and VI neurons. In addition, brains were stained using Hoechst stain to perform nuclei density and width analysis of the auditory cortical areas where dendrites were sampled. Finally, overall measurements of cortical thickness were acquired to determine whether there was a gross volumetric change in the AC.

**Figure 4:**
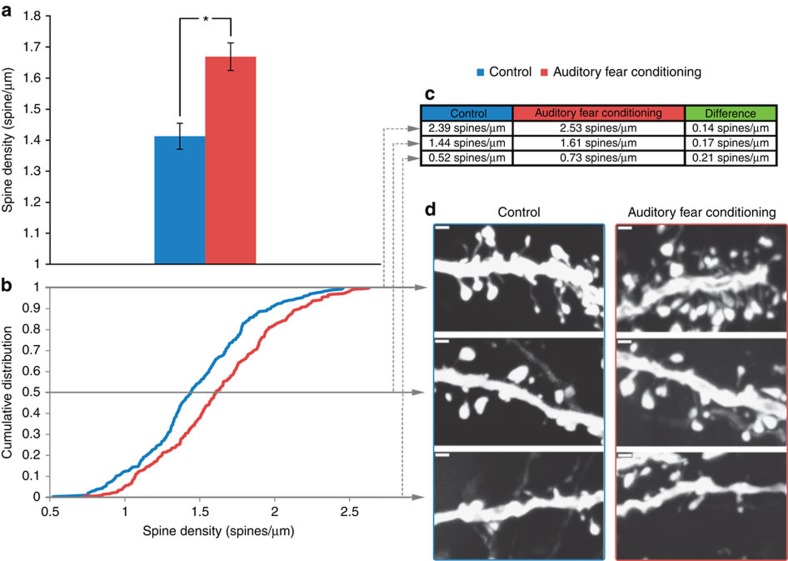
Increased dendritic spine density in the AC. (**a**) The bar graph shows the significant difference in average spine density between the auditory fear conditioning group and control from the same brains used for VBM analyses (*t*-test, *n*=9 auditory fear conditioning, *n*=9 control, *P*=0.013, *t*_16_=2.79). (**b**)The cumulative distribution of the average spine densities for each dendrite length shows a significant rightward shift (higher spine densities across the distribution) for the auditory fear-conditioned group (K–S test, *n*=290 and 300 for auditory fear-conditioned and control group respectively, *P*<0.0005, *D*=0.185). (**c**,**d**) The maximum, mid-point and minimum densities of the distribution for the auditory fear conditioning group and control are presented in the table (**c**) with commensurate confocal images of representative spine densities (**d**). Data are presented as mean±s.e.m. *P≤0.05. scale bars=1 micrometer.

Figure 4a represents the comparison of overall dendritic spine density measurements, treating each mouse (as opposed to each dendrite) as an independent sample. Dendritic spines in the AC for the auditory fear-conditioned group were 18% more dense than the control group *(t*-test, *n*=9 auditory fear conditioning, *n*=9 control, *P*=0.013, *t*_16_=2.79). A cumulative density analysis of the average dendritic spine density per dendritic length (Fig. 4b) revealed a rightward shift in the cumulative distribution (Kolmogorov–Smirnov (K–S) test, *n*=290 and 300 dendrites for auditory fear-conditioned and control group respectively, *P*<0.0005, *D*=0.185). Representative dendritic spine densities are presented in Fig. 4c, corresponding with the minimum, maximum and mid-point values. These data show that the auditory fear conditioning group has increased dendritic spine density relative to the control group.

**Figure 5:**
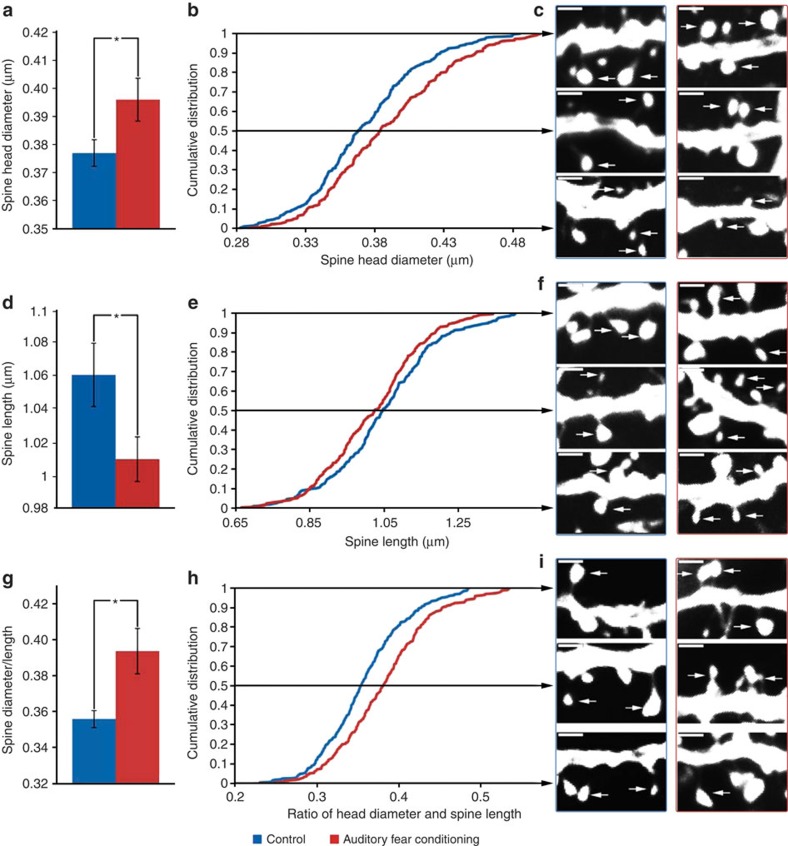
Shifts in dendritic spine width, length and the ratio. (**a**) The bar graph shows the significant difference in average spine diameter between the auditory fear conditioning group and control (*t*-test, *n*=9 auditory fear conditioning, *n*=9 control, *P*=0.040, *t*_16_=2.23). (**b**)The cumulative distribution of the average spine head diameter for each dendritic length shows a significant rightward shift (wider spine diameter across the distribution) for the auditory fear conditioning group when compared with controls (K–S test, *n*=290 and 300 for auditory fear-conditioned and control group, respectively, *P*≤0.0005, *D*=0.182). (**c**) The maximum, mid-point and minimum diameters from the distribution for the auditory fear conditioning and control groups are shown in representative confocal images. (**d**–**f**) These figures show the same analysis as those of spine diameter but for the length of the spines (*t*-test, *n*=9 auditory fear conditioning, *n*=9 control, *P*=0.045, *t*_16_=2.17) and (K–S test, *n*=290 and 300 for auditory fear-conditioned and control group, respectively, *P*≤0.021, *D*=0.123). These tests reveal that the auditory fear conditioning group has significantly shorter dendritic spine lengths than the control. The bottom panel shows the same panel for the ratio of the spine diameter and length (*P*≤0.05 for the overall comparison and *P*≤0.0001 for the K–S analysis). (**g**–**i**) These figures show the same analysis as those of spine diameter and length but for the ratio of the diameter to the length of the spines (*t*-test, *n*=9 auditory fear conditioning, *n*=9 control, *P*=0.01, *t*_16_=2.92) and (K–S test, *n*=290 and 300 for auditory fear-conditioned and control group, respectively, *P*≤0.0005, *D*=0.2178). These tests reveal that the auditory fear conditioning group has dendritic spines with a significantly larger ratio of head diameter to length when compared with controls. Data in bar graphs are presented as mean±s.e.m. Data in the line graphs are the average of the noted measure (head diameter, length and the ratio) per dendritic length presented as a cumulative distribution. Dendritic spine images are from maximum projections used in the analysis ( × 100). *P≤0.05.

With an analogous approach, Fig. 5a presents the overall average for spine head diameter, while Fig. 5b reports the cumulative distribution function. Notably, the dendritic spines from the auditory fear condition group were 5% wider when compared with control mice (*t*-test, *n*=9 auditory fear conditioning, *n*=9 control, *P*=0.040, *t*_16_=2.23), with a significant rightward shift (wider spine diameter across the distribution) for the auditory fear conditioning group when compared with controls (K–S test, *n*=290 and 300 for auditory fear-conditioned and control group, respectively, *P*≤0.0005, *D*=0.182). Figure 5c depicts dendritic spine heads in the range of the maximum, minimum and mid-point diameters observed between groups. Similarly, Fig. 5d–f reports the dendritic spine length between the groups. In particular, auditory fear conditioning shortens dendritic spines (5%), further substantiated by a leftward shift in the cumulative distribution curve (*t*-test, *n*=9 auditory fear conditioning, *n*=9 control, *P*=0.045, *t*_16_=2.17; K–S test, *n*=290 and 300 for auditory fear-conditioned and control group respectively, *P*=0.021, *D*=0.123).

Figure 5g–i reports the ratio of head diameter to dendritic spine length. Note that the two groups have a relatively similar distribution of dendrite spines with smaller ratios (<0.3), which then quickly diverges with the auditory-conditioned group having significantly higher ratio values. The divergence reflects the wider but simultaneously shorter dendritic spines of the auditory fear-conditioned group. The shorter but wider morphology may reflect more mature spines being found in the AC of auditory fear-conditioned mice. On average, the auditory-conditioned group has a 10.6% greater spine head diameter to length ratio compared with the controls *(t*-test, *n*=9 auditory fear conditioning, *n*=9 control, *P*=0.010, *t*_16_=2.92; K–S test, *n*=290 and 300 for auditory fear-conditioned and control group respectively, *P*≤0.0005, *D*=0.2178). Overall, these findings are consistent with shorter but wider spines following auditory fear conditioning.

Supplementary Figure 1 reports the same analysis of dendritic spine density, head width and length but for layer V pyramidal neurons located in the rhinal cortex, a cortical area not associated with auditory learning and for which we did not see a VBM change, selected for control analysis. Notably, there were no significant differences in the overall analysis of density, height, width or the height–width ratio in these rhinal cortex neurons. Further, there were no significant differences in the K–S analysis of the cumulative distribution of these metrics either.

### Nuclei density and width

**Figure 6:**
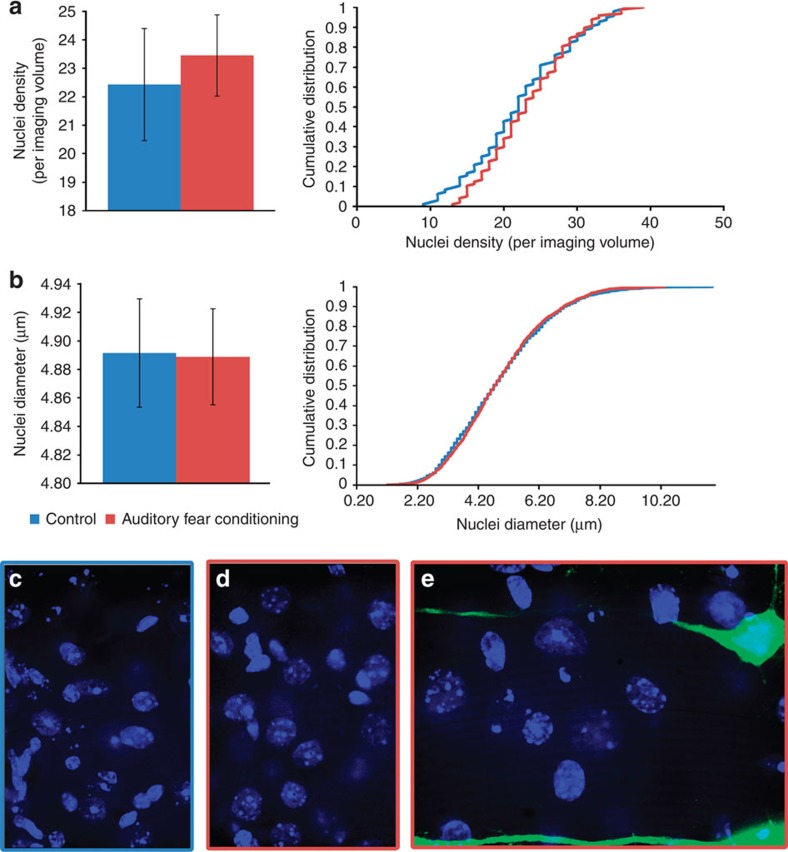
No measureable differences in nuclei density or width. (**a**- left) The comparison of the nuclei density between the auditory fear conditioning and control groups did not show a significant difference (*t*-test, *n*=8 per group, *P*=0.68, *t*_14_=0.422). (**a**-right) Further, the respective cumulative distribution of the cell nuclei density measures also did not show a significant difference (K–S test, *n*=96 per group, *P*=0.68, *D*=0.1046). (**b**-left) Likewise, there was an overall lack of a significant difference between the auditory fear conditioning group and the control group for nuclei diameter (*t*-test, *n*=8 per group, *P*=0.42, *t*_14_=0.830). (**b**-right) In addition, the cumulative distribution of nuclei diameters was not significantly different across groups (K–S test, *n*=2,042 for auditory fear conditioning, *n*=1,828 for control, *P*=0.45, *D*=0.0269). (**c**–**e**) The pictures show representative images of the Hoechst staining for the control group (**c**), auditory fear conditioning group (**d**) and a combined overlay with the *Thy*1-YFP-expressing neurons (**e**). Data in bar graphs are presented as mean±s.e.m. Data in the line graphs are the average of the noted measure (for example, density or width).

Given the hypothesis that increases in either neuron or glial count may account for the increased signal detected with VBM, we addressed this question using Hoechst dye to stain the nuclei of all the cells. The density of nuclei is used as a mechanism for counting all cells (including neurons and glial cells), whereas the size of the nuclei can be used as an approximate proxy measure for neurons and glial cells (glial cells generally have smaller nuclei^29^). Images were collected from the same subset of brains used for the dendritic spine analyses from the same regions where dendritic spines were imaged. Using previously published and validated counting methods, we assessed nuclei density, as well as the diameter of those nuclei^30^. These results are presented in Fig. 6a which shows no differences in nuclei density or the distribution of nuclei density after fear conditioning (*t*-test, *n*=8 per group, *P*=0.68, *t*_14_=0.422 and K–S test, *n*=96 per group, *P*=0.68, *D*=0.1046). Likewise, Fig. 6b shows that there was an overall lack of a significant difference between groups for nuclei diameter or the distribution of diameters after fear conditioning (*t*-test, *n*=8 per group, *P*=0.42, *t*_14_=0.830 and K–S test, *n*=2,042 for auditory fear conditioning, *n*=1,828 for control, *P*=0.45, *D*=0.0269). The images shown in Fig. 6c–e are representative images of the Hoechst staining for the control group, auditory fear conditioning group and a combined overlay with the *Thy*1-YFP-expressing neurons, respectively. Together, these data suggest that there is no difference in the nuclei density or nuclei width between groups. Further, as discussed below, it suggests that the VBM signal changes, while associated with spine density and morphology, are not associated with marked changes in cell density, across the range of cell sizes, including neuronal populations with large nuclei and glial populations with smaller nuclei.

### Layers V and VI pyramidal neuron diameter

In addition to changes in the dendritic spine density and morphology, the use of the *Thy1*-YFP line also afforded for the ability to measure changes in the layers V and VI neuron soma diameter. Supplementary Fig. 2a shows the division of layers V and VI of the AC. Supplementary Fig. 2b,c shows the overall analysis of the layer V neuron diameter and the respective distribution analysis, neither of which were significantly different between the experimental and control groups (*t*-test, *n*=9 per group, *P*=0.669, *t*_16_=0.435 and K–S, *n*=1,665 control and *n*=1,757, *P*=0.222, *D*=0.036). Analogously, Supplementary Fig. 2d,e shows the same analysis for the layer VI neurons, again with no significant differences between the two groups (*t*-test, *n*=9 per group, *P*=0.935, *t*_16_=0.082 and K–S, *n*=1,303 control and *n*=1,515, *P*=0.991, *D*=0.016). These data suggest that auditory fear conditioning is not associated with a specific change in layers V or VI neuronal soma size. Likewise, these data also suggest that changes in neuronal soma size are not associated with VBM signal.

### Cortical thickness

In addition to the investigations of the morphology of the neurons of the AC, analysis was also conducted on the thickness of the AC. On the basis of the data presented in Supplementary Fig. 3b, there is no significant difference in auditory cortical thickness between the auditory fear-conditioned and control groups (*t*-test, *n*=9 per group, *P*=0.46, *t*_16_=0.756). However, as shown in Supplementary Fig. 3c, there was a significant difference in the cumulative distribution of the cortical thicknesses, with the auditory fear conditioning group showing a greater number of measures above 1,250** **μm (K–S test, *n*=162 for auditory fear conditioning, *n*=159 for control, *P*=0.009, *D*=0.1808). Overall, these data suggest that while there may be an increase in density of shorter but wider dendritic spines, there is not necessarily a gross increase in the thickness of the cortex at least at the levels of our measurement. Interestingly, analysis of the distribution of the AC thickness did suggest that the auditory fear-conditioned group did have a preponderance of wider cortices at the upper end of the distribution.

### Correlation of VBM and confocal findings

**Figure 7:**
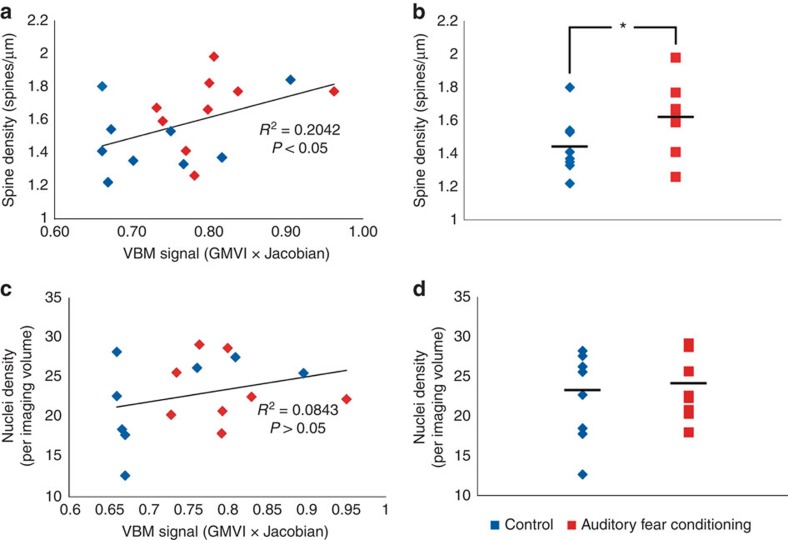
A positive correlation between VBM signal and dendritic spine density. (**a**) A scatter plot of the total data showing an overall significant correlation of spine density values to the VBM signal used for comparison between the auditory fear-conditioned group and the control group (Pearson's correlation, *n*=9 per group, one-way analysis, *P*=0.027, *R*^2^=0.2042). (**b**) A plot of the difference in dendritic spine density for the 18 animals used for the analysis (*t*-test, *n*=9 auditory fear conditioning, *n*=9 control, *P*=0.040, *t*_16_=2.23). (**c**)The overall correlation between the nuclei density and VBM signal was not significant (Pearson's correlation, *n*=8 per group, one-way analysis, *P*=0.138, *R*^2^=0.0843). The respective data used for the correlation is parsed in the top right and bottom right graphs. (**d**) A plot of the difference in nuclei density for the 16 animals used for the analysis (*t*-test, *n*=8 per group, *P*=0.68, *t*_14_=0.422).

While the VBM and confocal microscopy findings provide compelling evidence for changes in brain structure after exposure to a fear conditioning protocol, we sought to further quantify the relationship between each mode of examination with correlational analysis. To assess the relationship we covaried the average VBM signal in the AC used for permutation testing by FSL (grey matter voxel intensity multiplied by the Jacobian of the warp field) with the corresponding spine density, head diameter and length metrics for the auditory fear-conditioned group and the control group. The analysis in Fig. 7a shows a significant (*P*=0.027) and moderate positive correlation (*r*=0.46) between the VBM signal and dendritic spine density. In other words, increased VBM signal was associated with increased dendritic spine density. The correlations with spine head diameter, dendritic spine length and the diameter/length ratio were *r*=0.12, −0.29 and 0.24, although none reached significance (*P*=0.318, 0.122 and 0.169, respectively). Figure 7c represents the correlational analysis of grey matter voxel intensity multiplied by the Jacobian and the nuclei density per imaging volume. The correlation was *r*=0.29 and not significant (*P*=0.122). Together, these data show that there is a moderate correlation between VBM signal and dendritic spine density with auditory fear conditioning, highlighting this as a plausible mechanism of learning-induced VBM signal changes.

## Discussion

The large VBM literature in human subjects and the smaller but growing literature using animal models have highlighted the utility of MRI-based techniques to address meaningful questions regarding brain structure and function^31^,^32^. These reports must, however, only speculate as to the underlying cellular changes that may explain shifts in VBM signal. Here we assessed many of the major hypotheses in the field regarding the mechanism underlying VBM signal changes—shifts in dendritic spine density and character, shifts in cellular density (often attributed to glia) and shifts in neuronal size. The evidence presented supports the first hypothesis that VBM signal is coupled with regional dendritic spine density. Specifically, auditory fear conditioning increased VBM signal in the AC (on the order of 16–18%) of mice, and this was associated with an 18% increase in dendritic spine density, a 5% increase in spine head diameter and a 5% shortening of spine length. This contrasts with the VBM and confocal analysis of the rhinal cortex, which served as the control region, and revealed no such differences. Further, we found no evidence supporting the hypothesis that VBM signal changes reflect a proliferation of glia or neurons (that is, no differences in nuclei density) or reflect a change in neuronal soma size (that is, no changes in the layers V and VI neuron somas). In addition, there was no significant difference in the cortical thickness of the AC, suggesting that the local changes within the makeup of the AC do not necessarily result in an outward expansion in the AC.

It is interesting to note that the results support the idea that mice undergoing auditory fear conditioning had dendritic spines that were wider, but shorter in the AC. This is particularly provocative because dendritic spine morphology is closely associated with spine maturity and the likelihood that the spine contains an active synapse^33^. Immature spines are generally characterized by a long, filopodia-like shape with a narrow head. These filopodic^34^,^35^ dendritic spines are extremely dynamic in their environment, and some will stabilize—at which point they shorten and widen—forming a ‘mushroom-shaped' dendritic spine (for example, shorter but wider), reflecting a more mature, synapse-containing state. For example, previous work has shown that LTP can induce increases in dendritic spine width and decreases in length^36^. Thus, it is then possible that denser, shorter and wider dendritic spines following auditory fear conditioning reflect a maturation of dendritic spines in the AC. This in turn would support an overarching hypothesis that cortical areas play an important role in long-term memory^37^, and that the VBM learning-dependent signal may directly reflect these structural changes.

**Figure 8:**
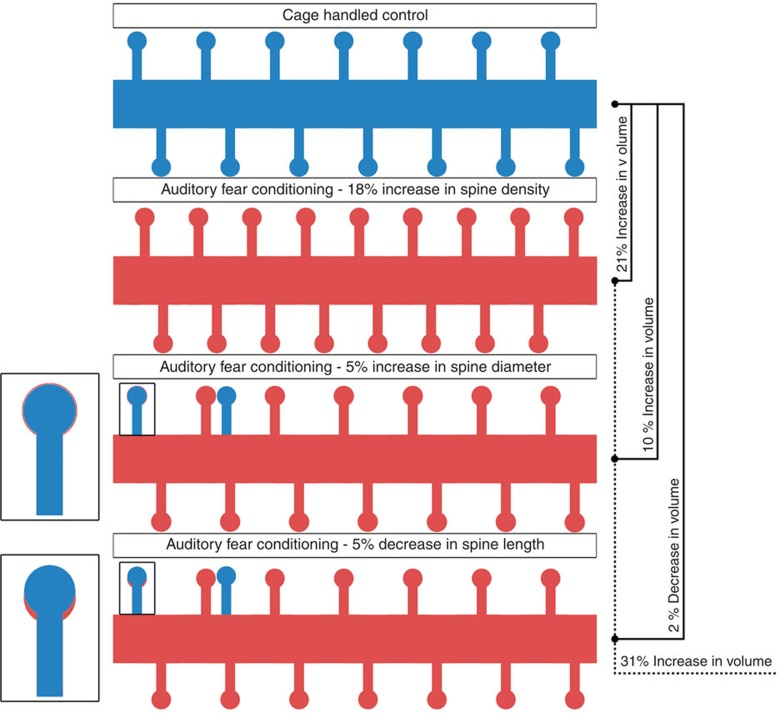
Schematic representation of volume changes with dendritic spine changes. (**a**-top row) A schematic representation of the control group's average dendritic width, length and density (scaled to represent a 10-μm length of dendrite). By assuming a spine to be a sphere atop a cylinder, we are able to approximate the impact of changes of density and morphology on overall volumetric changes at the macro scale. (**b**-second row) The representation of an auditory dendrite shows an 18% increase in density of spines only (keeping all other variables constant), which results in a commensurate 21% increase in volume. (**c**-third row) The second red dendrite shows a 5% increase in the diameter of spines holding all of the variables constant—the result is a 10% increase in volume above baseline. (**d**-bottom row) Examining the third and final red dendrite, with a 5% decrease in length of a spine, there is commensurate loss of only 2% of the volume. Changing all three variables, to reflect the totality of our findings here, results in a 31% increase of volume occupied by spines of the auditory fear conditioning group versus the control group. Representative magnifications of a single spine are shown next to the second and third red dendrite to get a sense of the effect of 5% difference.

Further considering the VBM imaging, it should be noted that the voxel sizes are on the order of 100 × 100 × 100 μm^3^. To detect signal changes at this level, it may be surprising that dendritic spine density can account for 20% of the variance of the signal. However, Fig. 8 demonstrates the plausibility of such an assertion. The volumetric (assuming a dendritic spine is a cylinder with a sphere on top) increases with respect to increased spine density and increased head diameter far outweigh that of decreasing the neck lengths alone. Further, the change in spine density alone leads to an increase in overall spine volume on the order of 21%; if combined with both the wider diameter heads and shorter lengths, the gross volumetric change is in the order of 31%. Considering a simplified and idealized case, if the density of neurons is ∼90 × 10^3^ neurons per mm^3^ with an approximate total dendritic length of 1,000 μm(refs 38, 39), then there are 90 neurons per voxel and 90,000 μm of total dendritic length. In turn, this would yield ∼126,000 dendritic spines in control mice and 153,000 dendritic spines in the auditory fear conditioning group. Therefore, the dendritic spines in auditory-conditioned mice will occupy ∼8,100 μm^3^, compared with 6,170 μm^3^ for the controls, an overall increase in ∼31% of neuronal dendritic spine volume. If we assume there is a commensurate increase in the pre-synaptic side (synaptic boutons), that would double the volumetric increase. Note that as part of the analysis, we are assuming that the increased volume of spines fills in previously ‘empty' space, therefore not necessarily leading to expansion or contraction of the AC, but rather changes to the density of spines within a particular volume of grey matter. Such an assertion could account for shifts in GMD without shifts in the thickness of the cortex as seen in the data presented here.

Importantly, shifts in the dendritic spine density are likely not the whole story and further consideration must be paid towards other potential structural changes that can co-occur. For example, it is also plausible that there is an increase in the dendritic arborization and axonal branching patterns that co-occur with increases in dendritic spine density changes^27^. One could also imagine that the plastic changes to the AC may also be accompanied by angiogenesis in the same vein as exercise induced angiogenesis of the motor cortex^40^. In addition, it should be pointed out here that although we did not see any shifts in the distribution of nuclei diameter or density in the AC, these were used as indirect measures of neuronal and glial densities. Future work could explicitly label these entities with the appropriate markers and ensure that there are no changes in the density or size of neurons, microglia, oligodendrocytes and astrocytes. Likewise, it is known that oligodendrocytes, microglia and astrocytes may be involved in plasticity and may be also expand in size, although we would have expected a commiserate change in nuclei diameter that would have been detected in our metrics^41^,^42^, we did not explicitly stain and measure microglia, astrocyte and oligodendrocyte size and morphology, which should be addressed in future work.

Considering the other areas that showed either significant or trending significant results, existing literature suggests similar patterns of dendritic density changes with fear conditioning. The VBM results presented here showed significant increases in the VBM signal in several nuclei of the amygdala (MeA, BLA and LA, and CeA) with auditory fear conditioning. Are these areas also associated with long-term changes in dendritic spine density? Using auditory fear conditioning, Heinrichs *et al*.^43^ reported that neurons in the BLA were associated with an increase in second- and third-order dendritic spine density (on the order of 20–30%) with auditory fear conditioning when compared with naive controls. These results were echoed by Pignataro *et al*., who examined spine density changes with context conditioning and auditory fear conditioning and reported, in both cases, that dendritic spine density increased in the BLA and hippocampus (the hippocampus is discussed further below).

The VBM signal change within the insula also is consistent with the literature in examining a role for insula, particularly in the pain pathway component of Pavlovian fear conditioning, and within fear-related disorders in humans such as post-traumatic stress disorder (PTSD) and social phobia^44^,^45^,^46^. Finally, we and others have demonstrated differential amygdala–insula functional connectivity as a function of gene pathways associated with PTSD and differential fear learning^47^.

With regards to the ACC, IL and PL, there are a few studies looking at dendritic spine densities in these areas that may account the inconsistent VBM findings. Restivo *et al*. have shown that there is a difference in the dendritic density in the ACC at 36 days after contextual fear conditioning, but not 24 h. Interestingly, the per cent change in dendritic spine density was slightly smaller than that in the AC (∼16% for apical dendrite spine density)^48^. This suggests at least two potential explanations for our ACC findings. The first is that a dendritic spine density change ∼16% maybe at the cusp of detectability with our VBM methodology. Further, since we only waited 14 days in our behaviour paradigm before euthanizing the animals, it is possible that our time table was too short, and waiting 36 days would result in more consistent and significant results in the ACC. Our *a priori* selection of the IL and PL was based on the growing literature that suggests a regulatory role in fear conditioning and extinction^49^,^50^. Yet interestingly, neither the PL nor the IL showed significant change in VBM signal with auditory fear conditioning. The discrepancy may reflect two aspects worthy of consideration. First, for the IL cortex, Vetere reported that contextual fear conditioning increased spine density and head size in the ACC and IL cortex when compared with pseudo-conditioned animals. The per cent change in dendritic spine density for these areas was on the order of 10–15% change in spine density in apical dendrites in layers II/III (ref. 51). Clearly, similar to the ACC results reported above, this per cent difference in dendritic spine density was smaller than found here in the AC, and in addition, this was in a different layer of cells. Perhaps, more interestingly, other studies have also suggested a *decrease* in dendrite terminal branches in the IL with repeated stress (similar to our 5 day training paradigm)^52^. Thus, a decrease in the number of dendrites and a smaller percentage increase in dendritic spine density with auditory fear conditioning could explain a lack of consistent VBM findings in the IL. With regards to the PL, there is no definitive study on the changes of dendritic spine density with auditory fear conditioning so we are unable to draw any conclusions.

While we did not initially investigate the hippocampus in the VBM analysis, we did a follow-up analysis based on the results of the work by Restivo , and did not identify VBM signal changes in the hippocampus. However, that follows from the results by Restivo that, similar to Pignataro *et al*.^53^, showed spine density changes in the hippocampus 24 h after conditioning, but, importantly, not 36 days later. These findings, along with ours, suggests an important temporary increase of spine density in the hippocampus that later reverts (that is, our samples were collected 2 weeks after conditioning).

Overall, this study establishes the first evidence we are aware of for the hypothesis that dendritic spine density may be the cellular mechanism that underlies changes in brain structure that is detected by voxel-based morphometry. Follow-up work should focus on providing further evidence for this hypothesis, including experimental manipulations to establish causality. In addition, further work should analyse postsynaptic density, angiogenesis and endothelial proliferation, dendrite arborization patterns, and glial activation, density and morphology. More generally, we have shown that using MRI techniques on animal models is a very powerful translational tool, since many of the areas detected by VBM in our repeated auditory fear conditioning group parallel findings in human studies of psychiatric pathology (particularly those that are fear related, such as PTSD). Further work should focus on establishing a more direct causal relationship, test the directionality of effect (for example, seeing how reduction in spine density with extinction in the frontal association cortex affects the VBM signal^54^) developing the full translational utility of these techniques. Finally, this study highlights the technical feasibility of utilizing MRI imaging of animal models to better understand the biological and cellular underpinnings of MRI techniques—a tremendously needed effort to better understand the neurobiological basis for MRI studies.

## Methods

### Mice

All experiments were conducted on *Thy1*-YFP-expressing mice (B6.Cg-Tg HJrs/J-Thy1-YFP, Jackson Labs, Bar Harbor, Maine). All male mice were 8 weeks of age when the fear conditioning was initiated. After concluding the 5 days of training, the mice were returned to their normal housing conditions in the Yerkes National Primate Research Center for 2 weeks before they were deeply anaesthetized with ketamine and dexmedetomidine, and perfused with paraformaldehyde. The mice were housed with a 12-h light/dark cycle grouped in cages (≤5 mice per cage) with *ad libitum* access to food and water. All conditioning was conducted during the light half of the cycle during the same time of day. All procedures were approved by the Institutional Animal Care and Use Committee of Emory University.

### Auditory fear conditioning

Mice were conditioned to pair an auditory tone with a mild foot shock. To do so, we used the SR-Lab Startle-Response system (San Diego Instruments, San Diego, California). The mice were trained on five consecutive days, with each training day consisting of 10 trials of an auditory tone (12 kHz) presentation of 30 s co-terminating with a 0.50 s 1.0-mA foot shock with an intertrial interval of 60 s. The controls were handled analogously with the exception that the SR-Lab conditioning programme was not initiated during the time the mice were in the startle-response cradles, and thus they were not exposed to shocks or tones.

### Magnetic resonance imaging

All MRI scanning was conducted on a research dedicated Bruker 9.4 Tesla MRI Scanner (Bruker, Billerica, MA). All brains were scanned using our previously designed *ex vivo* procedure^2^,^14^. In brief, perfused mouse brains were embedded in a gadolinium agarose matrix and scanned at multiples of 9 with a T2 RARE sequence. T2-weighted images were first acquired at 0.081 × 0.081 × 0.162 μm^3^ resolution (TE=28.6 ms, matrix 1,024 × 512 × 65, 10 averages, scan time ∼14 h).

### Voxel-based morphometry analysis

The acquired mouse structural volumes were parsed into individual brain volumes (each of the six scans that contained nine brain volumes was cut into nine individual brain volumes). The structural data were then analysed with a modified version (the built-in version is optimized for human data, we replaced the human MNI template with a mouse template of our own creation) of FSL^55^ (http://fsl.fmrib.ox.ac.uk/fsl/fslwiki/FSLVBM, Oxford, UK), an optimized VBM protocol^56^ carried out with FSL tools^57^. First, structural images were brain extracted (from the minor background noise created by the gadolinium agarose matrix) and grey matter segmented before being registered to our mouse standard space using nonlinear registration. The resulting images were averaged and flipped along the *x* axis to create a left-right symmetric, study-specific grey matter template. Second, all native grey matter images were nonlinearly registered to this study-specific template and ‘modulated' to correct for local expansion (or contraction) owing to the nonlinear component of the spatial transformation. The modulated grey matter images were then smoothed with an isotropic Gaussian kernel with a s.d. of 0.25 mm. Finally, voxel-wise general linear model (GLM) was applied using permutation-based nonparametric testing, correcting for multiple comparisons across space. This correction is based on the permutation null distribution of the volume-wise maximum statistic and therefore accommodates any spatial correlations among the errors. Results were displayed as a gradient of trending significance (*P*=0.10) to significance (*P*<0.01).

### Dendritic spine density and morphology analysis

Once the mouse brains were imaged, they were placed in 30% sucrose solution overnight and then sectioned into 35-μm-thick coronal sections on a freezing microtome held at −18 °C. Sections were then mounted onto slides using Mowiol mounting medium (Mowiol 40–88, Sigma-Aldrich, St Louis, MO), and the identifying marks were covered to blind the experimenter. On the basis of the previous work^26^,^27^,^28^, nine mouse brains were selected from each group for confocal analysis. The AC was identified using anatomical landmarks (anterior to posterior section required the presence of the stria terminalis (section 75 of 132 on the Paul Allen Reference Atlas—the same as used for the VBM significant signal), the dorsal border corresponded to the level of the dentate gyrus of the hippocampus, the ventral border was at the level of the ventral portion of the lateral ventricle and fimbria, medial border was the corpus callosum and lateral border was the edge of the cortex. Likewise, the rhinal cortex (control region) was defined as the cortical area with a dorsal border corresponding to the level of the point of bifurcation of the external capsule and amygdala capsule. For each animal, at least 10 unobstructed secondary and tertiary apical dendritic segments (within 160 μm of the soma) running parallel to the surface of the section were imaged on a spinning disk confocal (VisiTech International, Sunderland, UK) on a Leica DM5500 B microscope (laser *λ*=488 nm; Leica, Solms, Germany). *z*-Stacks were collected with a × 100 1.4 numerical aperture objective using a 0.1 μm step size, sampling above and below the dendrite. After imaging, we confirmed at × 10 that the image was collected from the intended regions. The AC of both hemispheres was sampled equally for each animal (at least five dendrites from each side).

Collapsed *z*-stacks were analysed using FIJI—(‘Fiji is just ImageJ', http://fiji.sc/Fiji). Protrusions ≤4 μm were considered spines^58^ and counted. In addition, spine lengths (from the base of the dendritic spine to the tip) and head widths (at the widest point) were measured. If a spine bifurcated, only the longest arm was measured and counted. Individual planes were also evaluated to detect protrusions extending perpendicular to the collapsed *z*-stack. Total spine number for each segment was normalized to the length of the dendritic segment (≥20 μm but ≤25 μm) to generate density values. All scoring was conducted by a single blinded rater.

### Nuclei density/width analysis

Analysis of nuclei density/width was conducted in accordance with previous work^30^. In brief, in addition to collecting *z*-stacks of the Thy1-YFP dendrites, 12 stacks (six left and six right) of the Hoechst-stained neurons were also collected per brain (laser *λ*=350 nm). The Hoechst images were pre-processed by finding the centre of the *z*-stack (defined with respect to the portion of the *z*-stack containing nuclei), selecting the images that composed 5 μm around the centre (total *z*-stack depth of 10 μm) and creating a maximum projection across the stack. The no-count boundaries of the stack were the top image of the *z*-stack and the right and upper border of the collapsed *z*-stack.

### Cortical thickness/neuronal soma diameter analysis

Analysis of the cortical thickness and the neuronal soma diameter of the layers V and VI neurons utilized six images (three left and three right) of the *Thy1*-YFP neurons that were collected per brain ( × 4 objective, fluorescence *λ*=488 nm). For the cortical thickness, three measurements were made across the AC for each image using FIJI (‘Fiji is just ImageJ', http://fiji.sc/Fiji; Supplementary Fig. 3b). For the measurement of neuronal soma diameter, the same images were used and the maximum width of neurons in layers V and VI were measured for the AC as defined in the dendritic spine density and morphology section.

## Additional information

**How to cite this article:** Keifer, O.P. *et al*. Voxel-based morphometry predicts shifts in dendritic spine density and morphology with auditory fear conditioning. *Nat. Commun.* 6:7582 doi: 10.1038/ncomms8582 (2015).

## Supplementary Material

Supplementary Information Supplementary Figures 1-3
