# Supplementary material for: Voxel-based morphometry predicts shifts in dendritic spine density and morphology with auditory fear conditioning
Source: Nat Commun. 2015 Jul 7;6:7582. doi: 10.1038/ncomms8582 (PMC4506522; doi:10.1038/ncomms8582)
Supplement: Supplementary Information — Supplementary Figures 1-3 [file ncomms8582-s1.pdf]

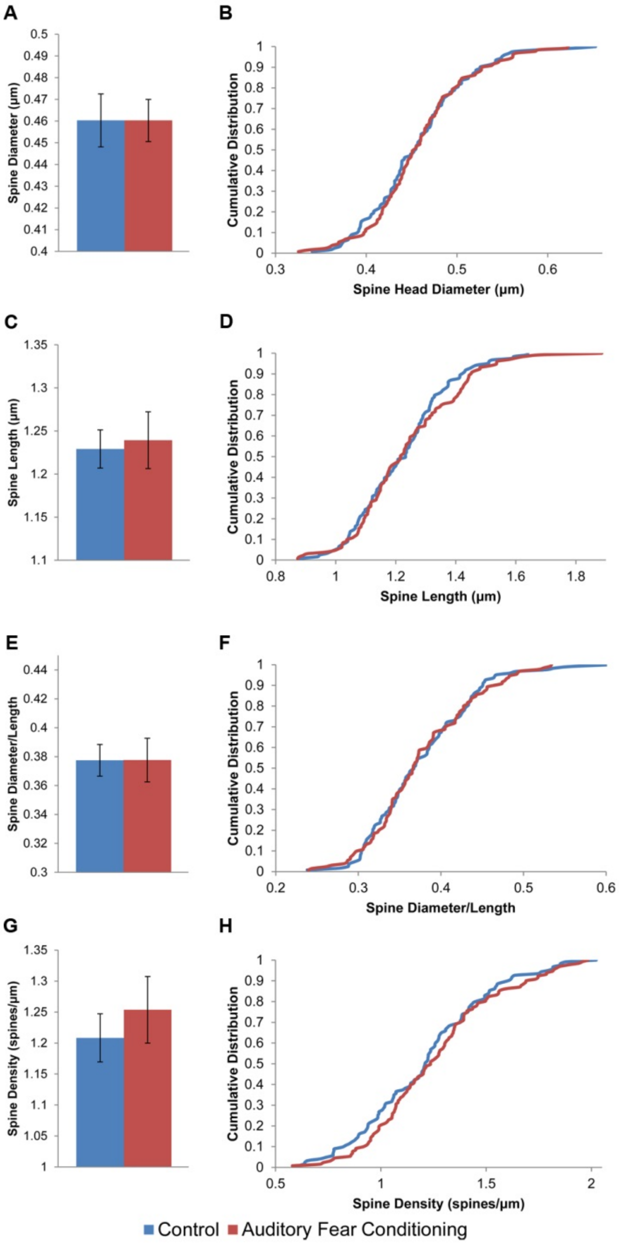

Supplementary Figure 1. Auditory Fear Conditioning Does Not Lead to Changes in Dendritic Spine Density, Width, Length, or the Ratio of Width to Length in the Rhinal Cortex.

(**a**) The bar graph shows no significant difference in average spine diameter between the auditory fear conditioning group and control (*t* test, n = 9 auditory fear conditioning, n = 8 control, P = 0.998, t_{15} = 0.002). (**b**)The cumulative distribution of the average spine head diameter for each dendritic length shows no significant differences between the auditory fear conditioning group when compared to controls (K-S test, n= 123 and 131 for auditory fear conditioned and control group respectively, P=0.871, *D* = 0.0735). (**c**) The bar graph shows no significant difference in average spine length between the auditory fear conditioning group and control (*t* test, n = 9 auditory fear conditioning, n = 8 control, P = 0.81, t_{15} = 0.246). (**d**)The cumulative distribution of the average spine length for each dendritic length shows no significant differences between the auditory fear conditioning group when compared to controls (K-S test, n= 123 and 131 for auditory fear conditioned and control group respectively, P=0.359, *D* =

0.1142). (**e**) The bar graph shows no significant difference in average ratio of the dendritic spine head diameter and length between the auditory fear conditioning group and control (*t* test, n = 9 auditory fear conditioning, n = 8 control, P = 0.99, t_{15} = 0.0072). (**f**)The cumulative distribution of the average spine length for each dendritic length shows no significant differences between the auditory fear conditioning group when compared to controls (K-S test, n= 123 and 131 for auditory fear conditioned and control group respectively, P=0.962, *D* = 0.0622). (**g**) The bar graph shows no significant difference in average dendritic spine density between the auditory fear conditioning group and control (*t* test, n = 9 auditory fear conditioning, n = 8 control, P = 0.52, t_{15} = 0.657). (**h**)The cumulative distribution of the average spine length for each dendritic length shows no significant differences between the auditory fear conditioning group when compared to controls (K-S test, n= 123 and 131 for auditory fear conditioned and control group respectively, P=0.569, *D* = 0.0969). Data are presented as mean ± s.e.m.

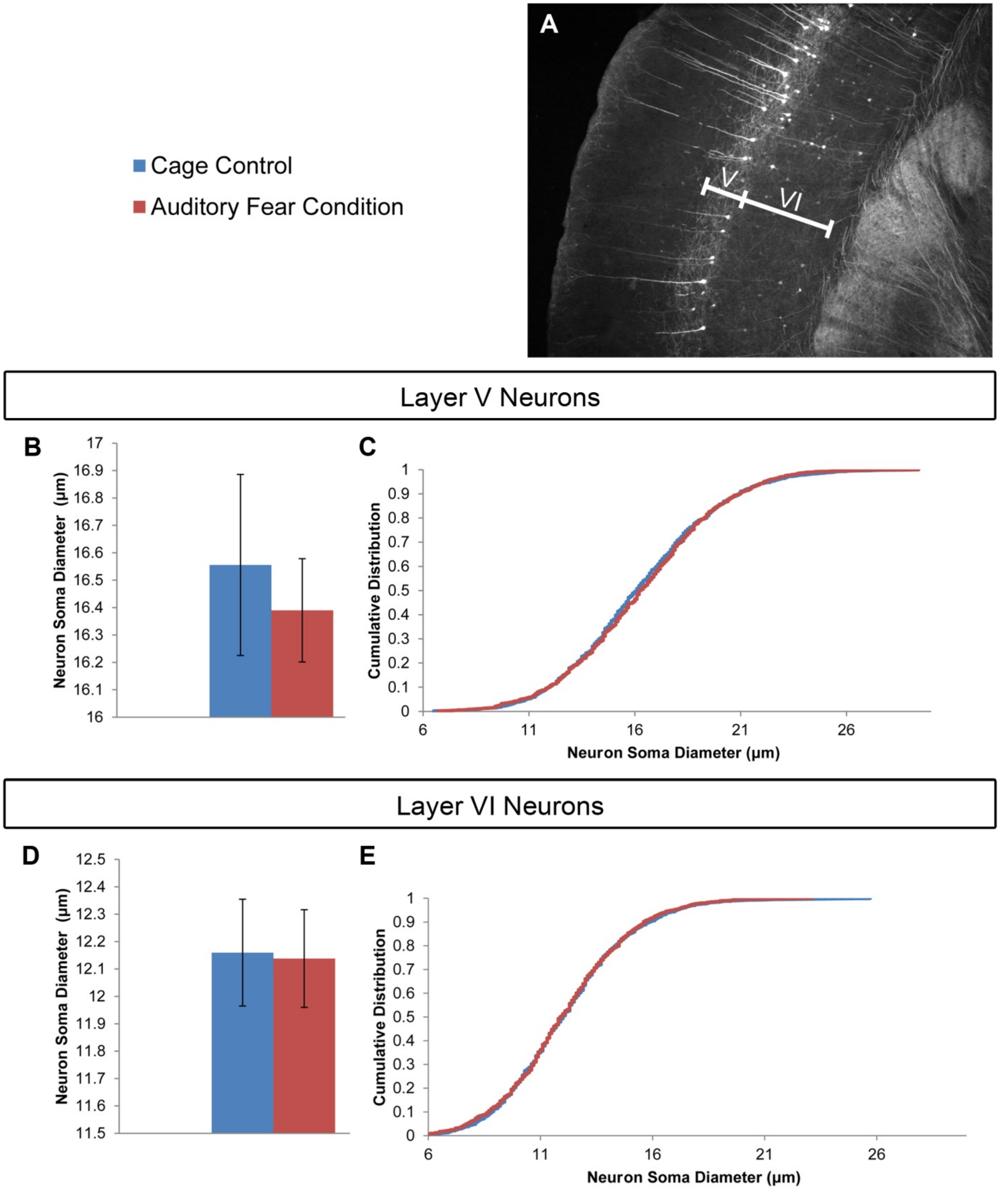

Supplementary Figure 2. There are No Significant Changes in Layer V and VI Neuronal Soma Diameter with Auditory Fear Conditioning
(**a**) Illustrative representative of how layer V and VI were defined based on the fluorescent pattern of the *Thy1*-YFP neurons (**b**) The bar graph shows no significant difference in average soma diameter for layer 5 neurons between the auditory fear conditioning group and control (*t* test, n = 9 for both groups, P = 0.669, t_{15} = 0.435). (**c**)The cumulative distribution of the average

soma diameter showed no significant differences between the auditory fear conditioning group when compared to controls (K-S Test, n = 1665 control and n = 1757, P = 0.222 D = 0.036).. Data are presented as mean ± s.e.m.

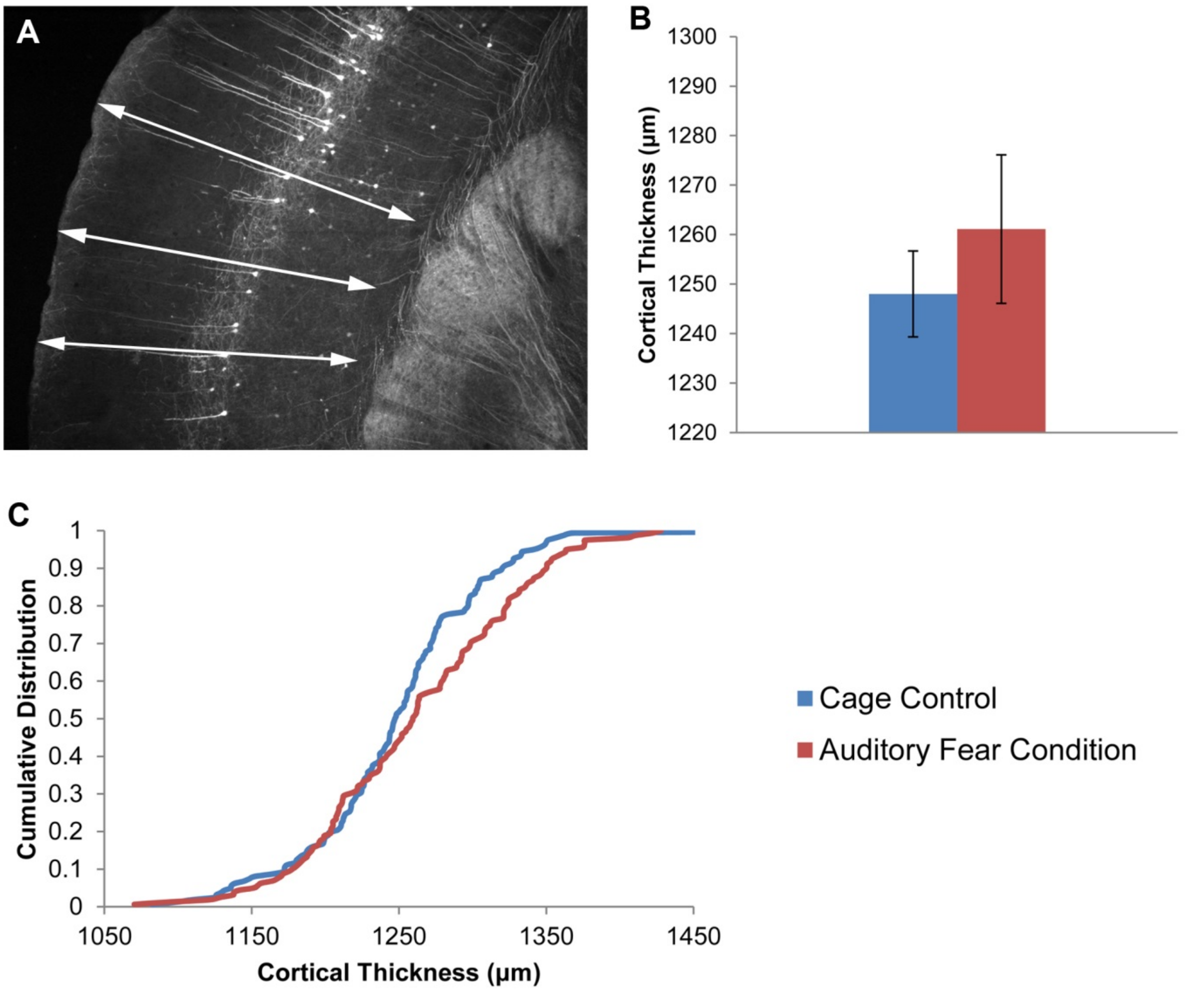

Supplementary Figure 3. There are No Significant Changes in Auditory Cortex Cortical Thickness with Auditory Fear Conditioning.

(**a**) Illustrative representative of the three measurements of the auditory cortex (**b**) The bar graph shows no significant difference in the average auditory cortex thickness between the auditory fear conditioning group and control ((t-test, n= 9 per group, P=0.46, t_{16} = 0.756). (**c**)The cumulative distribution of the cortex did show a significant difference between the auditory fear conditioning group when compared to controls (K-S test, n = 162 for auditory fear conditioning, n = 159 for control, P < 0.001, *D* = 0.1808). Data are presented as mean ± s.e.m.
